# Supplementary figures and images for: Effects of Sex and Cross-Sex Hormone Treatment on Renal MCT/SMCT Expression Following Prepubertal Gonadectomy
Source: Pharmaceutics. 2025 Feb 14;17(2):252. doi: 10.3390/pharmaceutics17020252 (PMC11859816; doi:10.3390/pharmaceutics17020252)

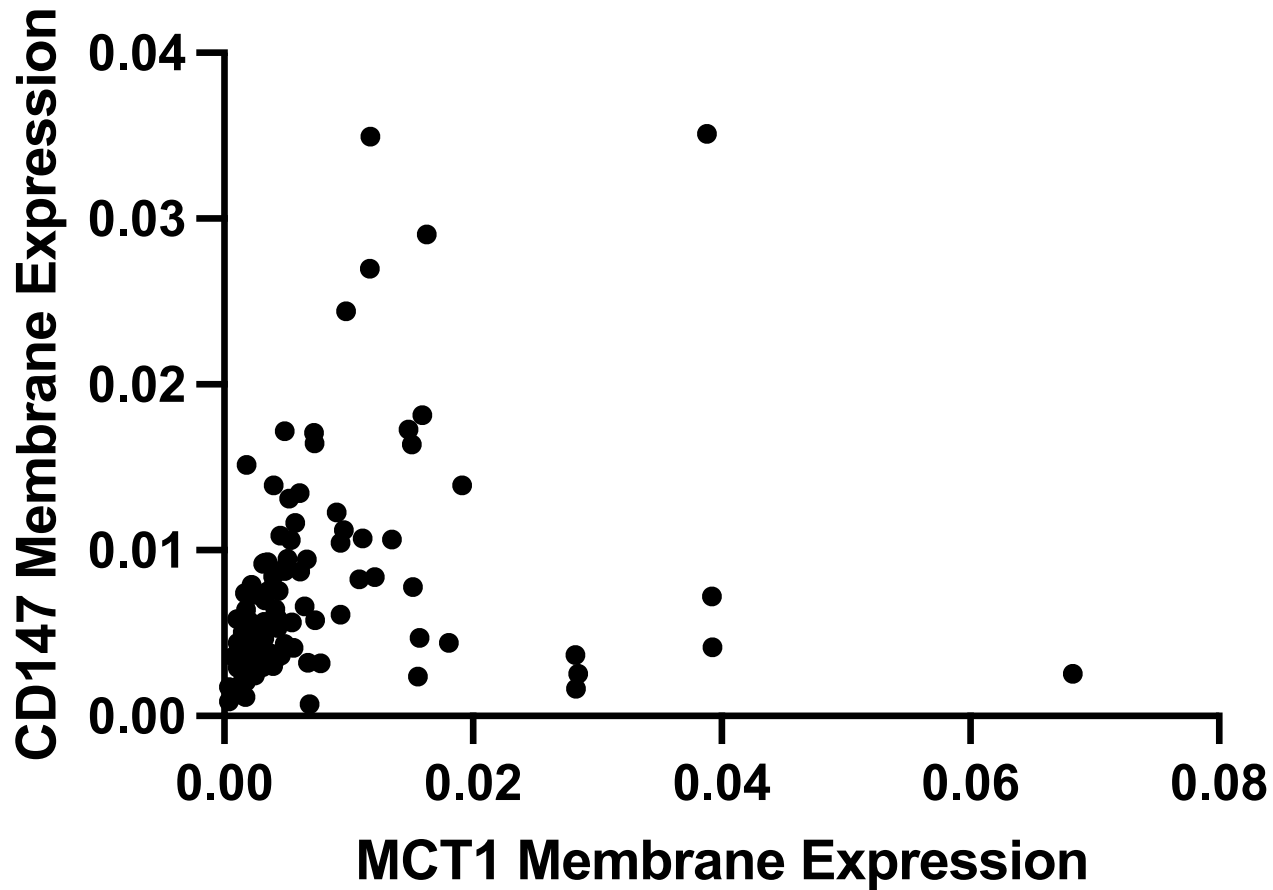

Supplement: Supplementary file 1 [file pharmaceutics-17-00252-s001.zip › Figure S1 Correlation MCT1 and CD147.pdf]

A

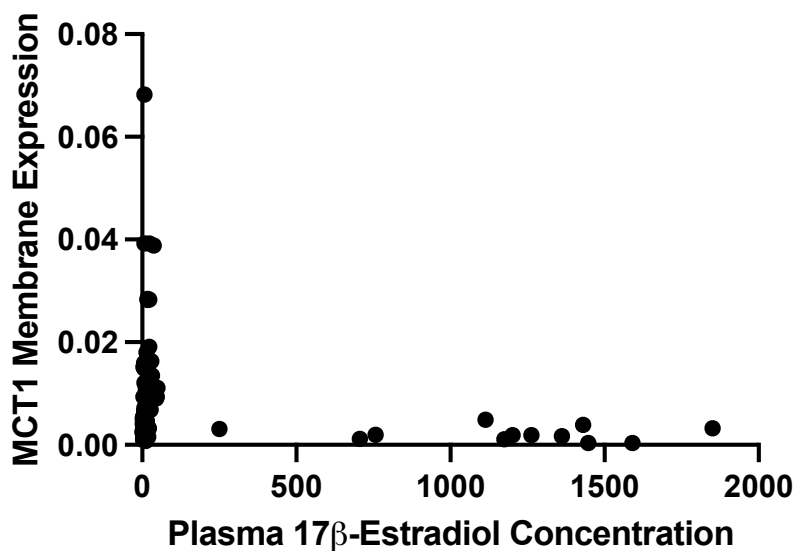

B

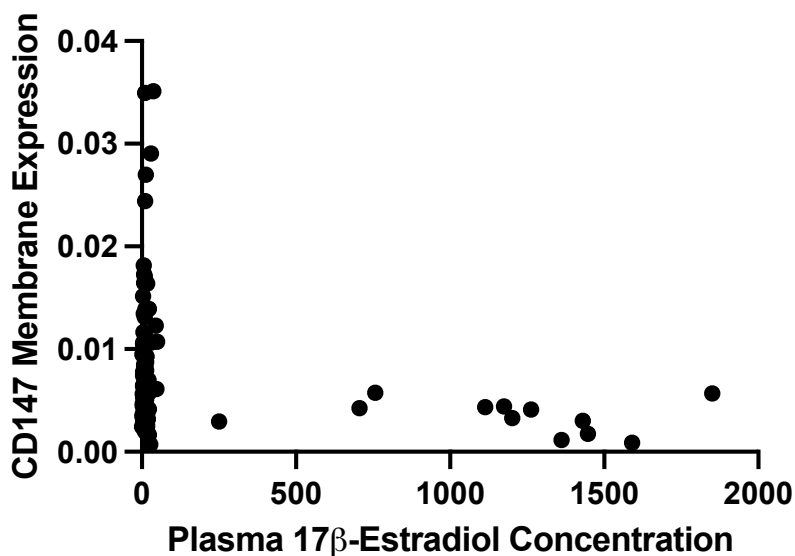

C

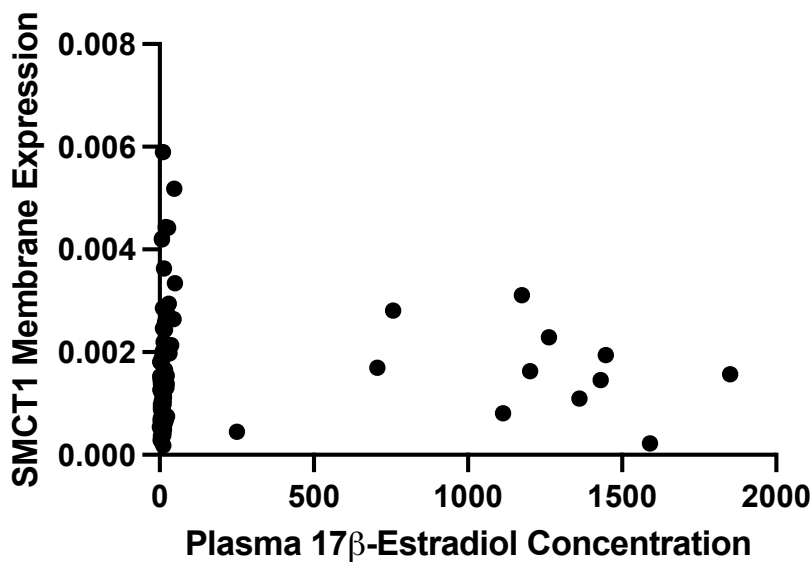

Supplement: Supplementary file 1 [file pharmaceutics-17-00252-s001.zip › Figure S2 Correlation Estradiol and Protein Expression.pdf]

A

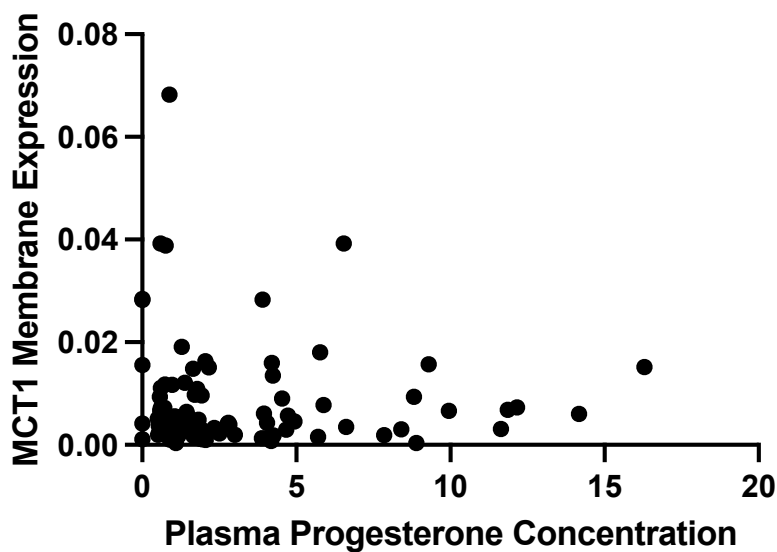

B

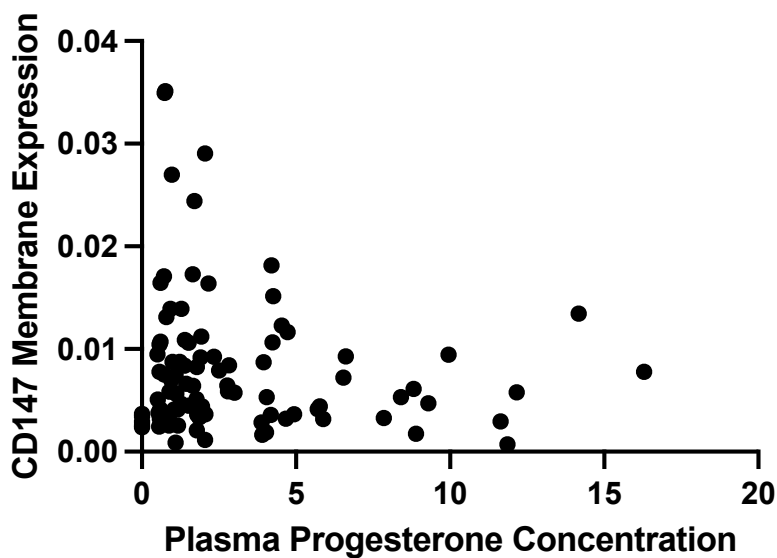

C

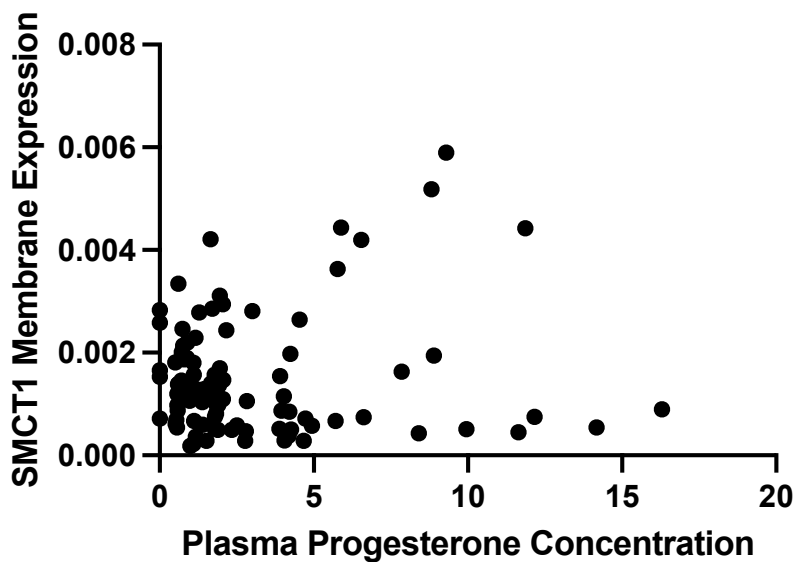

Supplement: Supplementary file 1 [file pharmaceutics-17-00252-s001.zip › Figure S3 Correlation Progesterone and Protein Expression.pdf]

A

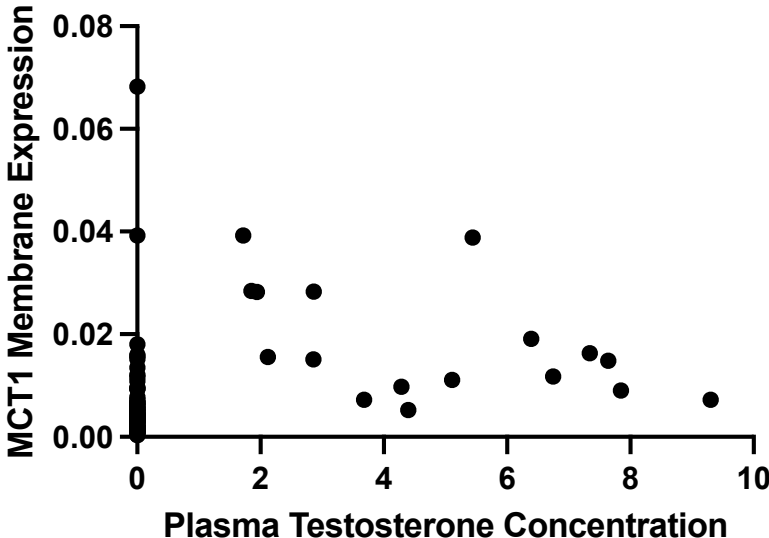

B

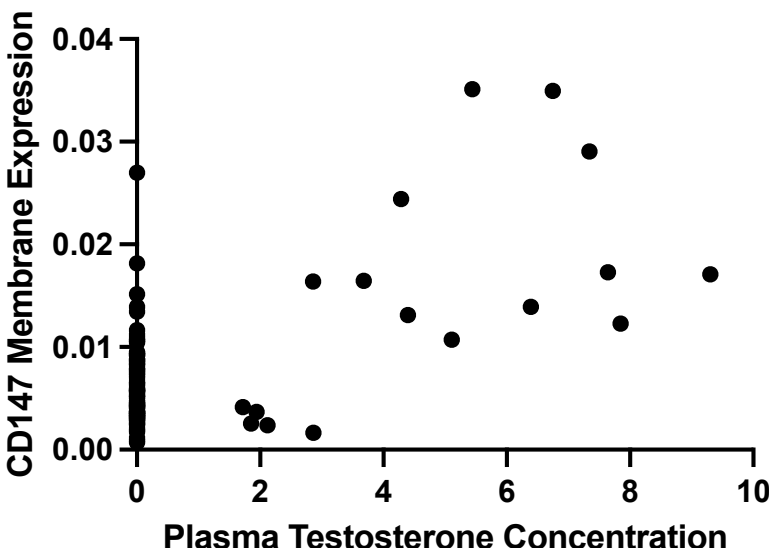

C

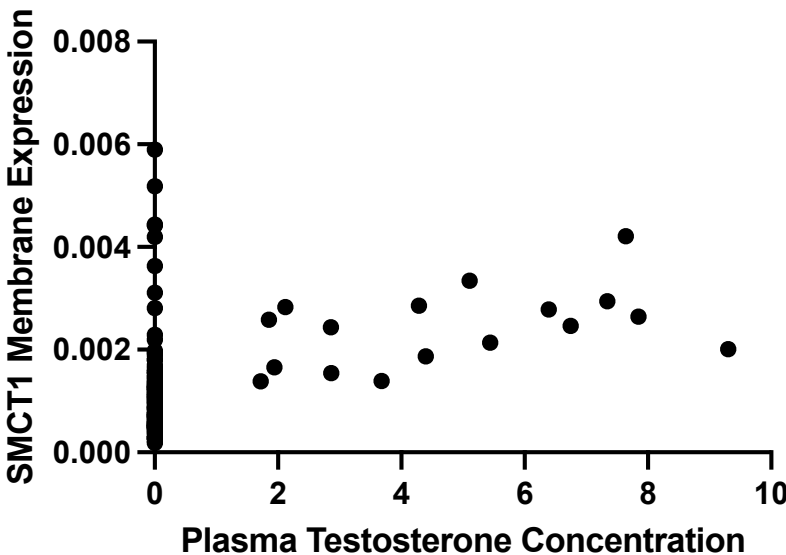

Supplement: Supplementary file 1 [file pharmaceutics-17-00252-s001.zip › Figure S4 Correlation Testosterone and Protein Expression.pdf]
